# High expression of a spliced variant of FKBP51 in peripheral blood mononuclear cells of melanoma patients may be related to PDL-1 on tumour and predictive of response to Ipilimumab

**DOI:** 10.1186/2051-1426-2-S3-P235

**Published:** 2014-11-06

**Authors:** Ester Simeone, Maria fiammetta Romano, Antonio Maria Grimaldi, Assunta Esposito, Marcello Curvietto, Miriam Paone, Giovanni Rinaldi, Gianluca Di monta, Massimiliano Di marzo, Federica Hauber, Lucia Festino, Anna D'Angelillo, Simona Romano, Stefania Staibano, Gennaro Ilardi, Rita Bisogni, Nicola Mozzillo, Paolo Antonio Ascierto

**Affiliations:** 1 Istituto Tumori di Napoli, Fondazione Pascale, Italy; 2 University of Naples, Federico II, Italy; 3 University of Catanzaro Magna Grecia, Italy; 4 Istituto Nazionale Tumori, Fondazione "G Pascale", Naples, Italy

## Background

Identifying molecular biomarkers in melanoma may provide useful diagnostic and therapeutic tools. Melanoma delivers immune suppressive stimuli through the pathway PDL-1/PD-1. Recent data suggest tumour-cell expression of PD-L1 in melanoma may be driven by constitutive oncogenic pathways. FK506 binding protein 51 (FKBP51), is an immunophilin that is highly expressed in melanoma and in tumour infiltrating lymphocytes (TIL). It is capable of immune suppression and has a relevant role in the progression of this tumour. We explored the expression of a variant generated by alternative splicing of FKBP51(FKBP51s) on TIL and its potential relation to PDL-1 in metastatic melanoma patients during Ipilimumab therapy (IPI).

## Methods

We collected melanoma samples (12 primaries and 64 metastases) and peripheral blood mononuclear cells (PBMC) of 76 metastatic melanoma patients at week 0,1,4,7,10 and 12 of IPI.

We measured expression levels of FKBP51s on TIL (range 7.0-36.0, arbitrary unit) and PDL-1 on tumour, by quantitative polymerase chain reaction (QPCR) and by immunohistochemical assay with a cutoff of 5%, respectively.

## Results

Expression of PDL-1 was found in 16% (2/12) and 40% (26/64) of primary and metastatic melanomas, respectively.

In melanoma negative for PDL-1, a low/no expression of FKBP51s was observed in TILs. In PDL-1 expressing melanomas, FKBP51s stained >80% of TILs. In addition, in melanoma negative for PDL-1, but with numerous PDL-1+ infiltrating macrophages, 10-15% of FKBP51s+ TILs was measured.

We found 29% of patients (22/76) had increased levels of FKBP51s (high FKBP51s) and PDL-1 positive, while 69% (53/76) had low levels (low FKBP51s) and PDL-1 negative at baseline (week 0). Indeed 11/22 (50%) of high FKBP51s responded to IPI and only 12/53 (23%) of low FKBP51s were responders (p < 0.01).

Furthermore, in responders patients we observed a decrease of the levels of FKBP51s during IPI therapy.

## Conclusions

We identified a spliced isoform of FKBP51 as a molecule associated to PDL-1, whose expression is increased in PBMCs of melanoma patients. High expression and reduction during IPI therapy may provide a potential predictive biomarker of response. These findings need more exploration.

## Consent

Written informed consent was obtained from the patient for publication of this abstract and any accompanying images. A copy of the written consent is available for review by the Editor of this journal.

